# The prevalence and outcome of short-acting β2-agonists overuse in asthma patients in Taiwan

**DOI:** 10.1038/s41533-021-00231-1

**Published:** 2021-04-20

**Authors:** Cheng-Yi Wang, Chih-Cheng Lai, Ya-Hui Wang, Hao-Chien Wang

**Affiliations:** 1 Department of Internal Medicine, Cardinal Tien Hospital and School of Medicine, College of Medicine, Fu Jen Catholic University, New Taipei City, Taiwan; 2 Department of Internal Medicine, Kaohsiung Veterans General Hospital, Tainan Branch, Tainan Taiwan; 3 Medical Research Center, Cardinal Tien Hospital and School of Medicine, College of Medicine, Fu Jen Catholic University, New Taipei City, Taiwan; 4 Department of Internal Medicine, National Taiwan University Hospital and College of Medicine, National Taiwan University, Taipei, Taiwan

**Keywords:** Asthma, Asthma

## Abstract

This study aims to investigate the prevalence of short-acting β2-agonist (SABA) overuse in asthma and the associated risk of acute exacerbation and mortality in Taiwan. We used the Taiwanese pay-for-performance asthma program database, which included patients aged between 12 and 100 years who were enrolled in the program between 2001 and 2015. Among a total of 218,039 patients, 34,641 (15.9%) patients are classified as SABA over-users. Compared with patients who did not receive inhaled corticosteroids (ICS) and collected ≤2 canisters, SABA over-users had a higher risk of severe exacerbations. SABA over-users had a higher risk of all-cause mortality compared with patients who did not receive ICS and collected ≤2 canisters. The overall prevalence of SABA overuse in Taiwan is 15.9%, and this is even higher in concomitant ICS users. In addition, the overuse of SABA is associated with an increased risk of severe exacerbation and death.

## Introduction

Asthma is a chronic inflammatory airway disease which affects about 339 million people worldwide^1^. Inhaled corticosteroid (ICS)-based medication can help to control asthma^2^, however it is not 100% effective and the control of asthma remains a great challenge for patients. Moreover, poorly controlled asthma could be associated with an increased risk of exacerbation, asthma-related hospitalizations, emergency department visits, and deterioration in lung function^3^. Before 2019, as-needed use of short-acting β2-agonists (SABAs) was recommended by the Global Initiative for Asthma (GINA) guidelines as a reliever medication and was traditionally prescribed for symptom relief by clinicians. However, increasing numbers of studies showed an increased risk of adverse events was associated with high SABA use, and the as-needed use of SABAs as relievers was replaced by ICS-formoterol in the updated GINA recommendations in 2019^4^.

The overuse of SABAs has become a serious concern but it is not easy to change overreliance on SABAs^5^. The SABA use In Asthma (SABINA) program was conducted to investigate global SABA and ICS use in asthma and their clinical consequences^6^. The first report in Europe showed that the prevalence of SABA overuse (at least 3 canisters per year) was 9% in Italy, 16% in Germany, 29% in Spain, 30% in Sweden, and 38% in the UK. The present study was conducted in Taiwan to understand the prevalence of SABA overuse and the associated risk of acute exacerbation and mortality in an Asian country.

## Results

### Baseline characteristics of asthma patients

Overall, a total of 218,039 patients were included in this study (Table 1). Among them, 34,641 (15.9%) patients were classified as having SABA overuse, of whom 19,350 (8.9%) collected 3 to 6 canisters and 15,291 (7.0%) collected ≥7 canisters. In this study, 156,653 patients had concomitant use of ICS while 61,386 did not. Among the concomitant ICS users, 16,358 (10.4%) collected 3 to 6 canisters and 13,275 (8.5%) collected ≥7 canisters. In contrast, among the patients who did not use ICS, only 2992 (4.9%) collected 3 to 6 canisters and 2016 (3.3%) collected ≥7 canisters. Overall, the prevalence of SABA overuse was higher among patients who received ICS compared with those without ICS (18.9% vs. 8.2%, *p* < 0.001). Among the patients who did not receive ICS, those who overused SABA were older, had a male prevalence, and more underlying comorbidities, such as myocardial infarction, congestive heart failure, peripheral vascular disease, cerebrovascular disease, peptic ulcer, renal disease, diabetes mellitus, liver disease and malignancies compared with those who were not overusing SABAs. A similar trend was observed for concomitant ICS users (Table 1).

**Table 1:** Baseline characteristics of asthma patients on ICS therapy in different SABA use groups.

	Without ICS use and no. of SABA canisters			With ICS use and no. of SABA canisters		
	0–2 (*n* = 56,378)	≥3 (*n* = 5,008)	*p* value	0–2 (*n* = 127,020)	≥3 (*n* = 29,633)	*p* value
	*N* (%)	*N* (%)		*N* (%)	*N* (%)	
Age, mean (SD)	42.1 (19.1)	49.8 (20.1)	<0.0001	45.1 (18.5)	50.6 (19.0)	<0.0001
Age categories			<0.0001			<0.0001
12–19	8,886 (15.76)	463 (9.25)		12,995 (10.23)	1,859 (6.27)	
20–39	19,002 (33.7)	1,197 (23.9)		40,999 (32.28)	7,446 (25.13)	
40–59	16,697 (29.62)	1,610 (32.15)		42,169 (33.2)	9,952 (33.58)	
≥60	11,793 (20.92)	1,738 (34.7)		30,857 (24.29)	10,376 (35.02)	
Gender						
Female	34,332 (60.9)	2,904 (57.99)	<0.0001	76,561 (60.27)	16,470 (55.58)	<0.0001
Comorbidities						
Myocardial infarction	162 (0.29)	39 (0.78)	<0.0001	440 (0.35)	221 (0.75)	<0.0001
Congestive heart failure	1,034 (1.83)	309 (6.17)	<0.0001	2,695 (2.12)	1,488 (5.02)	<0.0001
Peripheral vascular	309 (0.55)	50 (1)	<0.0001	808 (0.64)	233 (0.79)	0.0042
Cerebrovascular	1,070 (1.9)	160 (3.19)	<0.0001	2,705 (2.13)	1,017 (3.43)	<0.0001
Rheumatologic disease	690 (1.22)	64 (1.28)	0.7392	1,638 (1.29)	503 (1.7)	<0.0001
Peptic ulcer	6,830 (12.11)	761 (15.2)	<0.0001	16,191 (12.75)	5,026 (16.96)	<0.0001
Renal disease	1,339 (2.38)	166 (3.31)	<0.0001	2,894 (2.28)	1,085 (3.66)	<0.0001
DM	4,905 (8.7)	486 (9.7)	0.0161	11,264 (8.87)	3,688 (12.45)	<0.0001
liver disease	3,657 (6.49)	256 (5.11)	0.0001	8,513 (6.7)	2,216 (7.48)	<0.0001
tumor	743 (1.32)	194 (3.87)	<0.0001	1,991 (1.57)	678 (2.29)	<0.0001

During the 1-year baseline period, the collection of SABA and SAMA both as a monotherapy and in combination, LABA and LAMA monotherapy were more frequent among patients who overused SABA compared with those not over using SABA. In addition, SABA over-users had a significantly higher severity of asthma compared with non-SABA over-users (*P* < 0.001; Table 2).

**Table 2:** Clinical classification of asthma severity according to the National Asthma Education and Prevention Program (NAEPP) guidelines.

	Without ICS use and no. of SABA canisters			With ICS use and no. of SABA canisters		
	0–2 (*n* = 56,378)	≥3 (*n* = 5,008)	*p* value	0–2 (*n* = 127,020)	≥3 (*n* = 29,633)	*p* value
	*N* (%)	*N* (%)		*N* (%)	*N* (%)	
Medications						
ICS	0 (0)	0 (0)	—	39,983 (31.48)	11,359 (38.33)	<0.0001
LABA	1,481 (2.63)	269 (5.37)	<0.0001	5,210 (4.1)	2,492 (8.41)	<0.0001
LABA_ICS	0 (0)	0 (0)	—	98,552 (77.59)	23,968 (80.88)	<0.0001
LAMA	181 (0.32)	75 (1.5)	<0.0001	959 (0.75)	1,017 (3.43)	<0.0001
SABA	9,857 (17.48)	4,248 (84.82)	<0.0001	34,995 (27.55)	28,092 (94.8)	<0.0001
SABA_SAMA	605 (1.07)	1,217 (24.3)	<0.0001	3,091 (2.43)		<0.0001
SAMA	1,340 (2.38)	1,105 (22.06)	<0.0001	3,339 (2.63)	8,725 (29.44)	<0.0001
Systemic Beta Agonist	35697 (63.32)	3,674 (73.36)	<0.0001	81,167 (63.9)	22,268 (75.15)	<0.0001
Severity of asthma (NAEPP guidelines)			<0.0001			<0.0001
Intermittent	15,233 (27.3)	815 (16.41)		15,590 (12.8)	3,488 (12.47)	
Mild persistent	20,444 (36.6)	1,752 (35.29)		30,302 (24.8)	6,521 (23.31)	
Moderate persistent	17,669 (31.6)	2,066 (41.61)		63,708 (52.2)	14,118 (50.46)	
Severe persistent	2,521 (4.5)	332 (6.69)		12,467 (10.2)	3,849 (13.76)	

### The association between asthma exacerbation and SABA uses

**Fig. 1:**
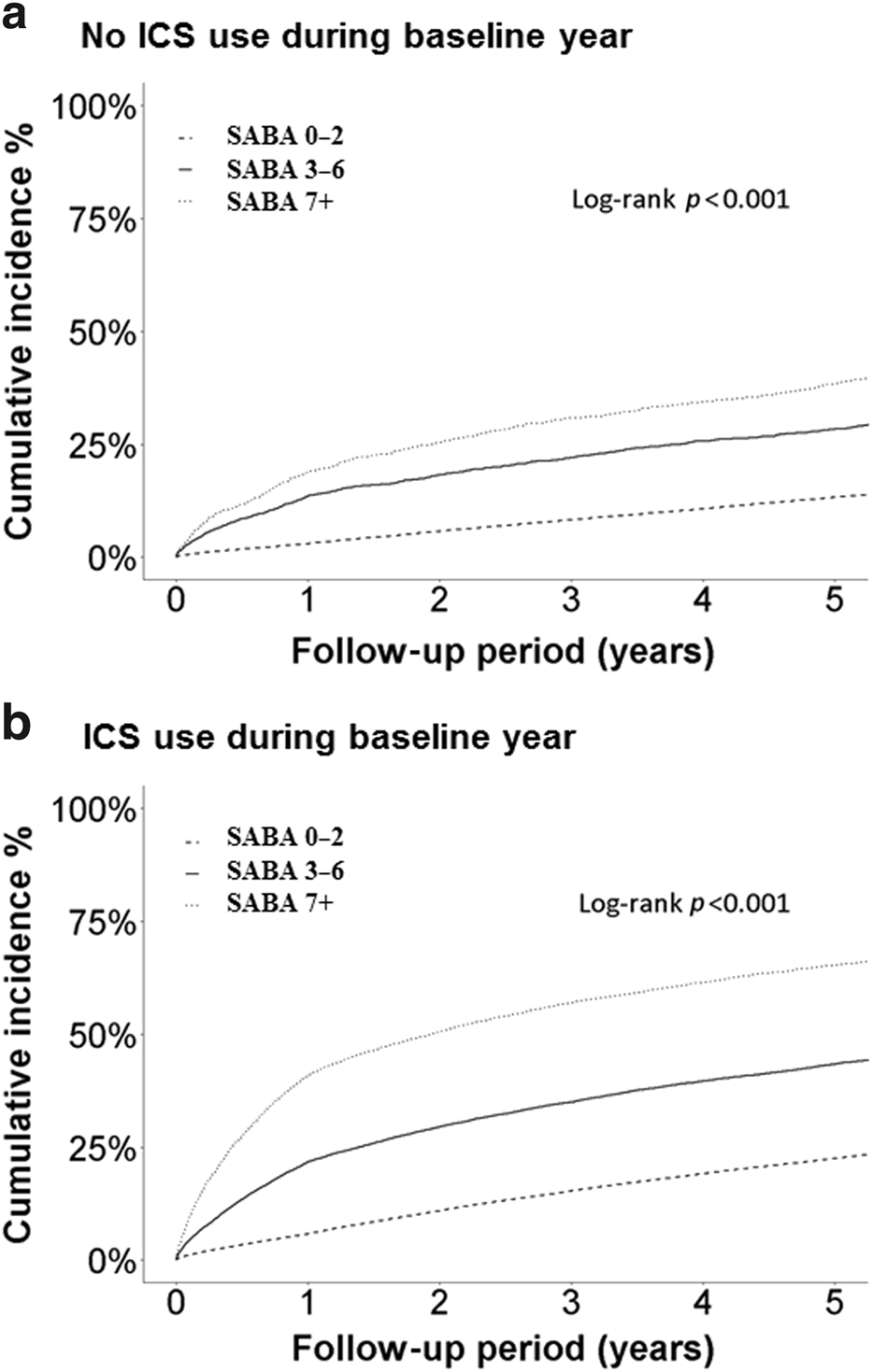
Incidence of asthma exacerbation. Kaplan–Meier plot of the incidence of asthma exacerbation by baseline SABA use among non-ICS uses (**a**) and ICS-uses (**b**).

Compared to patients who did not receive ICS and who collected ≤2 canisters, SABA over-users had a higher risk of severe exacerbation (Table 3). For both ICS and non-ICS users, more SABA use was associated with higher risk of asthma exacerbation (both *p* < 0.001, Fig. 1), and this trend did not differ according to the severity of asthma reported (Supplementary Figs. 1 and 2). Moreover, the risk was higher in patients who collected ≥7 canisters compared with those who collected 3 to 6 canisters. Overall, the highest risk was observed in patients who received concomitant ICS and ≥7 SABA canisters (adjusted HR, 4.94, 95% CI, 4.79–5.09). This trend was observed during both the 1-year baseline period and the follow-up period.

**Table 3:** Association between baseline ICS and SABA use and risk of severe exacerbation.

	Event	Person-year	IR^a^	Crude HR (95% CI)	Adjusted^b^ HR (95% CI)
Severe exacerbation during follow-up period					
No ICS and 0–2 SABA use	10,818	38,7419.55	2.79%	Reference	Reference
No ICS and 3–6 SABA use	973	17,230.47	5.65%	2.01 (1.89–2.15)	1.42 (1.33–1.51)
No ICS and ≥7 SABA use	1170	7579.32	15.44%	5.27 (4.97–5.6)	3.13 (2.95–3.33)
ICS and 0–2 SABA use	36,368	77,0087.41	4.72%	1.65 (1.62–1.69)	1.54 (1.51–1.58)
ICS and 3–6 SABA use	7723	83,212.24	9.28%	3.18 (3.09–3.28)	2.43 (2.36–2.5)
ICS and ≥7 SABA use	10,428	34,249.67	30.45%	9.83 (9.57–10.1)	4.94 (4.79–5.09)
Severe exacerbation during 1 year					
No ICS and 0–2 SABA use	1164	55,651.17	2.09%	Reference	Reference
No ICS and 3–6 SABA use	322	2803.35	11.49%	5.46 (4.83–6.18)	3.09 (2.72–3.49)
No ICS and ≥7 SABA use	680	1593.22	42.68%	19.93 (18.13–21.9)	7.92 (7.18–8.73)
ICS and 0–2 SABA use	5109	124,010.99	4.12%	1.97 (1.85–2.1)	1.85 (1.73–1.97)
ICS and 3–6 SABA use	2650	14,798.64	17.91%	8.48 (7.92–9.09)	4.69 (4.37–5.04)
ICS and ≥7 SABA use	7095	8833.41	80.32%	36.63 (34.42–38.97)	10.15 (9.47–10.87)

^a^IR, incidence rate.

^b^Adjusted HR: adjusted for age, gender, and asthma drug use.

### The association between all-cause mortality and SABA use

**Fig. 2:**
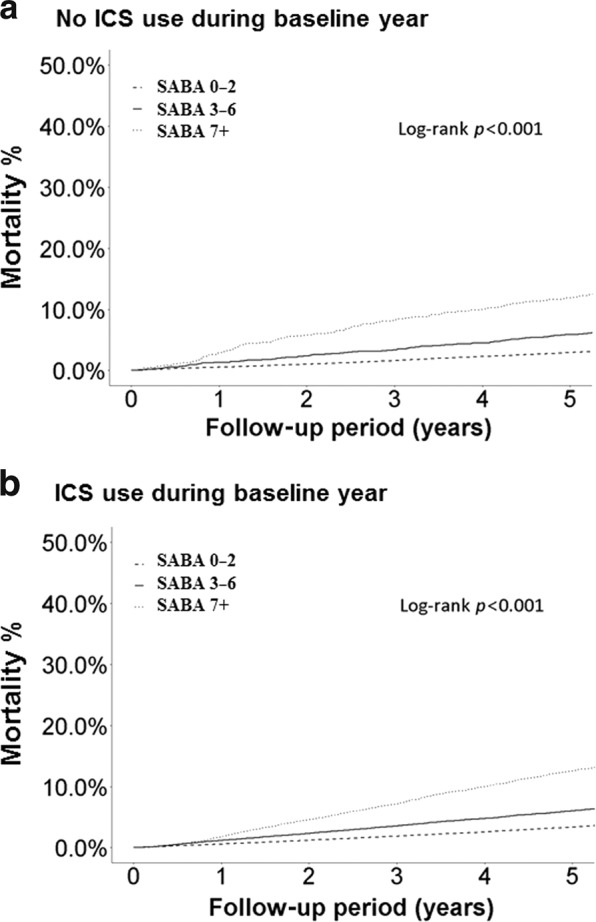
Rate of mortality. Kaplan-Meier plot of the rate of mortality by baseline SABA use among non-ICS uses (**a**) and ICS-uses (**b**).

Compared to patients who did not receive ICS and collected ≤2 canisters, SABA over-users had a higher risk of all-cause mortality (Table 4). For both ICS and non-ICS users, more SABA use was associated with higher mortality (both *p* < 0.001, Fig. 2), and this trend did not differ according to the severity of asthma reported (Supplementary Figs. 3 and 4). For patients who collected ≤2 canisters, ICS use was associated with a lower all-cause mortality compared with those who did not received ICS (adjusted HR, 0.82, 95% CI, 0.69–0.97) and this trend was observed in both the one-year period and during the follow-up period.

**Table 4:** Association between baseline ICS and SABA use and risk of all-cause mortality.

	Event	Person-year	IR^a^	Crude HR (95% CI)	Adjusted^b^ HR (95% CI)
All-cause mortality during follow-up period					
No ICS and 0–2 SABA use	2665	424298.42	0.63%	Reference	Reference
No ICS and 3–6 SABA use	222	21331.35	1.04%	1.68 (1.46–1.92)	1.17 (1.02–1.34)
No ICS and ≥7 SABA use	486	12273.75	3.96%	6.48 (5.88–7.13)	2.49 (2.25–2.75)
ICS and 0–2 SABA use	6329	900821.03	0.70%	1.13 (1.08–1.18)	0.95 (0.9–0.99)
ICS and 3–6 SABA use	1289	119990.6	1.07%	1.71 (1.6–1.83)	1.17 (1.09–1.25)
ICS and ≥7 SABA use	3090	87888.33	3.52%	5.63 (5.35–5.93)	2.01 (1.89–2.13)
1 year all-cause mortality					
No ICS and 0–2 SABA use	189	56272.18	0.34%	Reference	Reference
No ICS and 3–6 SABA use	20	2981.8	0.67%	2 (1.26–3.17)	1.18 (0.74–1.87)
No ICS and ≥7 SABA use	133	1947.17	6.83%	20.35 (16.3–25.41)	5.36 (4.22–6.8)
ICS and 0–2 SABA use	415	126805.18	0.33%	0.97 (0.82–1.16)	0.82 (0.69–0.97)
ICS and 3–6 SABA use	105	16305.18	0.64%	1.92 (1.51–2.43)	1.13 (0.89–1.45)
ICS and ≥7 SABA use	595	13009.84	4.57%	13.62 (11.57–16.04)	3.15 (2.59–3.81)

### Propensity score-matched cohort study

To minimize the effect of possible confounding factors, propensity score match method was applied to identify two similar subgroup of the patients who collected 0 to 2 and ≥3 canister of SABA per year. After pairwise matching (1:1), two subgroups with 24,261 patients in each shared the similar baseline characteristics in the previous one year including bronchodilator and ICS use, and the severity of asthma, were identified (Table 5). Similarly, overuse of SABA (≥3 canister per year) was associated with a higher risk of severe exacerbation and all-cause mortality than those use only 0 to 2 canister per year (Table 6).

**Table 5:** Demographic and clinical characteristics of the propensity score-matched population.

	Before propensity-score matched			After propensity-score matched		
	No. of SABA canisters			No. of SABA canisters		
	0–2 (*n* = 183,491)	≥3 (*n* = 34,548)	*p* value	0–2 (*n* = 24,261)	≥3 (*n* = 24,261)	*p* value
	*n* (%)	*n* (%)		*n* (%)	*n* (%)	
Age, mean (SD)	44.2 (18.7)	50.5 (19.2)	<0.0001	46.9 (18.7)	47.1 (18.8)	0.1383
Gender			<0.0001			0.5138
Male	72,543 (39.53)	15,229 (44.08)		10,169 (41.92)	10,240 (42.21)	
Female	110,948 (60.47)	19,319 (55.92)		14,092 (58.08)	14,021 (57.79)	
Charlson Score, mean (SD)	1.42 (1.00)	1.77 (1.35)	<0.0001	1.56 (1.12)	1.57 (1.13)	0.2564
Comorbidities						
Myocardial infarction	601 (0.33)	368 (1.07)	<0.0001	137 (0.56)	145 (0.6)	0.6328
Congestive heart failure	3971 (2.16)	2503 (7.24)	<0.0001	972 (4.01)	990 (4.08)	0.6783
Peripheral vascular disease	1196 (0.65)	355 (1.03)	<0.0001	198 (0.82)	205 (0.84)	0.7262
Cerebrovascular disease	3927 (2.14)	1334 (3.86)	<0.0001	678 (2.79)	675 (2.78)	0.9341
Dementia	974 (0.53)	438 (1.27)	<0.0001	184 (0.76)	196 (0.81)	0.5366
Rheumatologic disease	2408 (1.31)	547 (1.58)	<0.0001	330 (1.36)	328 (1.35)	0.9374
Peptic Ulcer disease	23,774 (12.96)	6179 (17.89)	<0.0001	3633 (14.97)	3735 (15.4)	0.1969
Hemiplegia or paraplegia	110 (0.06)	50 (0.14)	<0.0001	29 (0.12)	28 (0.12)	0.8946
Renal disease	4863 (2.65)	1464 (4.24)	<0.0001	736 (3.03)	760 (3.13)	0.5285
AIDS	63 (0.03)	13 (0.04)	0.7634	8 (0.03)	7 (0.03)	0.7962
Diabetes	17,540 (9.56)	4917 (14.23)	<0.0001	2689 (11.08)	2750 (11.34)	0.3801
Liver disease	12,073 (6.58)	2576 (7.46)	<0.0001	1667 (6.87)	1698 (7)	0.5796
Cancer	3201 (1.74)	1148 (3.32)	<0.0001	562 (2.32)	580 (2.39)	0.5899
Medications						
ICS	40,077 (21.84)	11,342 (32.83)	<0.0001	7810 (32.19)	7652 (31.54)	0.1237
LABA	6696 (3.65)	2761 (7.99)	<0.0001	1440 (5.94)	1441 (5.94)	0.9847
LABA/ICS	98,660 (53.77)	23,908 (69.2)	<0.0001	15,487 (63.83)	15,650 (64.51)	0.1228
LABA/LAMA	26 (0.01)	25 (0.07)	<0.0001	7 (0.03)	7 (0.03)	1
LAMA	1149 (0.63)	1089 (3.15)	<0.0001	331 (1.36)	366 (1.51)	0.1818
SABA	44,991 (24.52)	32,256 (93.37)	<0.0001	22,745 (93.75)	22,118 (91.17)	<0.0001
SABA/SAMA	3708 (2.02)	7969 (23.07)	<0.0001	2081 (8.58)	2704 (11.15)	<0.0001
SAMA	4680 (2.55)	9829 (28.45)	<0.0001	3375 (13.91)	3377 (13.92)	0.9791
Oral beta agonist	116,937 (63.73)	25,894 (74.95)	<0.0001	17,190 (70.85)	17,273 (71.2)	0.4062
Xanthine	119,182 (64.95)	28,258 (81.79)	<0.0001	18,579 (76.58)	18,683 (77.01)	0.2634
ICS dose						0.7232
No use	56,326 (30.7)	4988 (14.44)		4269 (17.6)	4203 (17.32)	
Low	94,313 (51.4)	14,292 (41.37)	<0.0001	11,064 (45.6)	11,067 (45.62)	
Medium	24,500 (13.35)	9782 (28.31)	<0.0001	6353 (26.19)	6351 (26.18)	
High	8352 (4.55)	5486 (15.88)		2575 (10.61)	2640 (10.88)	
Severity of asthma (NAEPP guidelines)						0.9544
Intermittent	30,837 (17.32)	4289 (13.06)		3041 (13.15)	3102 (13.42)	
Mild persistent	50,775 (28.52)	8244 (25.10)		6127 (26.50)	6148 (26.60)	
Moderate persistent	81,418 (45.73)	16,143 (49.14)		11,455 (49.54)	11,381 (49.24)	
Severe persistent	14,995 (8.42)	4174 (12.71)		2501 (10.82)	2481 (10.73)	

*ICS* inhaled corticosteroid, *SABA* short-acting beta-agonist, *SAMA* short-acting anti-muscarinic agent, *LABA* long-acting beta-agonist, *LAMA* long-acting anti-muscarinic agent.

**Table 6:** Association between baseline short-acting beta-agonist (SABA) use and risk of all-cause mortality and severe exacerbations.

	0–2 canisters of SABA			≥3 canisters of SABA			Crude HR (95% CI)	Adjusted^b^ HR (95% CI)
	Event	Person-year	IR^a^	Event	Person-year	IR^a^		
Before propensity-score matched								
All-cause mortality during follow-up period	8395	1,137,793.2	0.738%	4227	207,943.3	2.033%	2.57 (2.47–2.67)	1.16 (1.1–1.23)
All-cause mortality during 1 year	785	183,118.8	0.429%	672	34,193.6	1.965%	4.59 (4.14–5.08)	1.69 (1.44–1.99)
Severe exacerbation during follow-up period	35,742	997,819.0	3.582%	14,481	143,379.0	10.100%	2.73 (2.68–2.78)	1.36 (1.32–1.4)
Severe exacerbation during 1 year	7543	209,257.6	3.605%	5766	40,930.9	14.087%	4.39 (4.24–4.55)	1.67 (1.59–1.75)
After propensity-score matched								
All-cause mortality during follow-up period	1674	155,408.4	1.077%	2047	151,028.3	1.355%	1.19 (1.11–1.27)	1.17 (1.09–1.25)
All-cause mortality during 1 year	145	24,190.9	0.599%	278	24,123.3	1.152%	1.92 (1.57–2.35)	1.9 (1.56–2.32)
Severe exacerbation during follow-up period	6950	126,030.4	5.515%	8592	111,593.7	7.699%	1.37 (1.33–1.41)	1.39 (1.34–1.43)
Severe exacerbation during 1 year	1736	28,965.0	5.993%	2883	28,026.9	10.287%	1.71 (1.61–1.82)	1.72 (1.62–1.83)

^a^IR, incidence rate.

^b^Adjusted RR: adjusted for age, gender, and asthma drug use.

## Discussion

This nationwide study in Taiwan, was the first Asthma surveillance study carried out in Asia and it had several significant findings. First, we found that the overall prevalence of SABA overuse in Taiwan was 15.9%. Compared with European countries, the prevalence of SABA overuse in Taiwan was higher than Italy (9%), similar to Germany (16%) and lower than France (28.3%), Spain (29%), Poland (29–37%), Sweden (30%) and the UK (38%)^7–9^. The prevalence of patients who collected 3 to 6 canister per year in Taiwan was 4.9%, which was lower than many European countries, including Italy (6%), Germany (10%), Spain (19%), Sweden (25%), and the UK (24%). The prevalence of patients who collected ≥7 canisters per year in Taiwan was 3.3%, which was similar to Italy (3%), but lower than other countries, such as Germany (5%), Sweden (6%), Spain (10%) and the UK (15%).

The lower prevalence of SABA overuse in Taiwan compared with most of the European countries may be due to the implementation of the National Health Insurance system in Taiwan, which is a compulsory social insurance program. More than 99.9% of Taiwanese citizens have been enrolled in this program, so most patients can obtain cheap and efficient medical services, and most asthma patients can get and adjust their medication according to their physicians’ instructions. In addition, this study was based on a national P4P asthma-care program, so the rate of patients with appropriate asthma medication could be enhanced compared with the country-wide norm. However, even under this program, >15% of asthma patients overused SABA. Moreover, we found that the prevalence of SABA overuse was higher in concomitant ICS users (18.9%) compared with those who did not use ICS (8.2%). This may support the observation that patients often take their reliever medication (SABA) instead of their controller medication (ICS) when they have symptoms. The use of a fast-acting anti-inflammatory reliever (low dose ICS/formoterol combination as needed) wound be a better strategy for this patient behavior. In addition, overuse of SABA was more common in older patients, men, patients with comorbidities and concomitant use of other inhaled bronchodilators, such as SAMA, LABA and LAMA. These findings could lead to healthcare authorities paying more attention to these groups and developing appropriate policies to improve care for these asthma patients.

Second, we found that the overuse of SABA was associated with a higher risk of severe exacerbation and all-cause mortality. Additionally, we observed that the use of more SABA canisters per year was correlated with a higher risk of severe exacerbation and all-cause mortality. In order to make sure these results, we performed the propensity score-matched cohort study to minimize the effect of possible confounding factors. Propensity score match method was applied to identify two similar subgroup of the patients who collected 0 to 2 and ≥ 3 canister of SABA per year. Overuse of SABA (≥3 canister per year) was associated with a higher risk of severe exacerbation and all-cause mortality than those use only 0 to 2 canister per year. However, it should be kept in mind that propensity matching is unlikely to fully overcome confounding by severity, and that some confounding is still likely to be present.

These findings was consistent with the findings of the SABINA program in Sweden^10^. In the US, a study^11^ based on a Medicaid and a commercial insurance database had similar findings as it reported that the use of ≥3 SABA canisters per year was the best predictor for an increased risk of asthma-related exacerbations, and each additional SABA canister per year was associated with an 8 to 18% increase in the risk of asthma-related exacerbations. Another study^12^ also demonstrated that the use of >12 SABA canisters each year was associated with a higher risk of death. In fact, many studies^13,14^ have previously reported adverse effects associated with the regular or frequent use of SABAs, including β-receptor down-regulation, decreased bronchodilator response, decreased broncho-protection, rebound hyperresponsiveness, increased allergic response and increased eosinophilic airway inflammation. All of these findings indicate that the overuse of SABAs in asthma could be associated with adverse outcomes and should be avoided^15^.

Furthermore, we noted that ICS users had lower all-cause mortality compared with non-ICS users, irrespective of SABA use. This finding is reasonable because ICS remains the cornerstone of asthma management. In fact, many studies^16–19^ have demonstrated the benefits of ICS use for asthma patients. Even in mild, recent-onset asthma, once daily, low-dose budesonide can help decrease the risk of severe asthma-related events, reduce lung function decline, and improve symptom control^16^. All of these findings echo the recommendation by the GINA to clinicians that preferential use of ICS/formoterol reliever therapy maintenance a better way than SABA prn used for the management of asthma^20^.

This study had one major limitation: we used data from a P4P program database. Under this program, physicians were encouraged to treat asthma patients according to the guidelines and patient education could be enhanced. Therefore, it may not be possible to generalize the data to all other clinical settings. However, the way this program works could be utilized in other places to overall improve the quality of care for asthma patients. Additionally, we did not assess the differential risk with different SABA and the status of smoking in this study because the detail data was not available. Further study is warranted to investigated the effect of different SABAs. Finally, we can only obtain the data regarding of all-cause mortality, but no asthma specific mortality was available.

In conclusion, the prevalence of SABA overuse was about 16% in Taiwan, and even higher among concomitant ICS users. In addition, the overuse of SABA was associated with an increased risk of severe exacerbation and death. To better control asthma, healthcare authorities and clinicians need to reduce the overuse of SABAs.

## Methods

### Data source

Asthma is a common chronic disease for which patients require regular medical treatment and medication. This treatment is often self-managed in order to avoid the risk of acute exacerbations. Taiwan has implemented the pay-for-performance (P4P) asthma program, and encouraged medical institutions to join the program to strengthen tracking management and health education for asthma patients. Patients are only eligible to participate in the program if they were diagnosed with asthma at the same clinic or hospital at least twice within 90 days by the same doctor. The doctors also have to explain the purpose of the treatment plan to the patient and ask for their cooperation with regular return visits and follow-ups. The Asthma education program and asthma medication were given according to the Taiwanese asthma guidelines.

### Study population and SABA or other asthma-related medication exposure

The study population comprised patients aged 12 to 100 years old who were enrolled in the Taiwan P4P asthma program between 2001 and 2015. Patients were excluded for the following reasons: (1) age <12 or >100 years, (2) unknown demographic data, or (3) history of tuberculosis, bronchitis or other respiratory disease before entering the P4P asthma program. Consequently, the final study population comprised 218,039 patients.

When entering the Taiwan P4P asthma program, a 12-month baseline period was used as the period of exposure to SABA. We also calculated the usage of other asthma medications, including ICS, long-acting β2-agonists (LABA), LABA/ICS, long-acting muscarinic antagonist (LAMA), short-acting muscarinic antagonist (SAMA) and SABA/SAMA.

### Definition of SABA overuse

Overuse of SABA was defined as patients who collected ≥3 canisters per year. Outcomes including severe exacerbation and all-cause mortality were measured during the follow-up period which started after the final day of the 12-month baseline period. Severe exacerbations were defined as asthma-related hospitalizations or emergency department visits. Individuals were followed until death, emigration, or the end of the study.

### Statistical analysis

Descriptive statistics (mean, standard deviation, frequency and percentage) were used to characterize the study population at baseline. Baseline characteristics were compared between groups using Chi-squared tests for categorical variables and independent *t*-tests for continuous variables.

Cox regression models were used to calculate the crude and adjusted hazard ratios (HRs) of different outcomes in the two study cohorts. Adjusted HRs and 95% confidence intervals (CIs) were calculated using Cox regression models. The cumulative incidence of events was constructed using the Kaplan–Meier method and the differences between the two treatment groups were tested using the log-rank test. The crude incidence rate of different outcomes was calculated as the total number of events during the follow-up period divided by the person-years at risk. A *P*-value of <0.05 was considered to indicate statistical significance in all analyses. The software package used for data analysis was SAS version 9.4 (SAS Institute Inc., Cary, NC, USA).

### Propensity score-matched cohort study

All patients were divided into two subgroup: SABA-fair-use subgroup with SABA 0 to 2 per year and SABA-overuse subgroup with SABA ≥ 3 canisters per year. To minimize imbalances in baseline characteristic covariates between the two subgroups, we performed 1:1 propensity score matching. Covariates that may have caused interference or bias in the association between exposure and outcomes of interest such as demographic characteristics, comorbidities, medication, and asthma severity were included in the propensity matching.

### Ethics statement

All information from patient files was retrospectively and anonymously collected from medical reports, so no written informed consent was collected. No personal identifying information was collected. The Ethical Committee of National Taiwan University Hospital approved the research (#201812069RIPC).

### Reporting summary

Further information on research design is available in the Nature Research Reporting Summary linked to this article.

## Supplementary information

Supplementary Information

Reporting Summary

## Data Availability

The data that support the findings of this study are available on request from the corresponding author. The data are not publicly available because the use of the National Health Insurance Research Database is limited to research purposes only.

## Code availability

Available upon request.
