# Supplementary material for: The prevalence and outcome of short-acting β2-agonists overuse in asthma patients in Taiwan
Source: NPJ Prim Care Respir Med. 2021 Apr 20;31:19. doi: 10.1038/s41533-021-00231-1 (PMC8058069; doi:10.1038/s41533-021-00231-1)
Supplement: Supplementary file 1 — Supplementary Information [file 41533_2021_231_MOESM1_ESM.pdf]

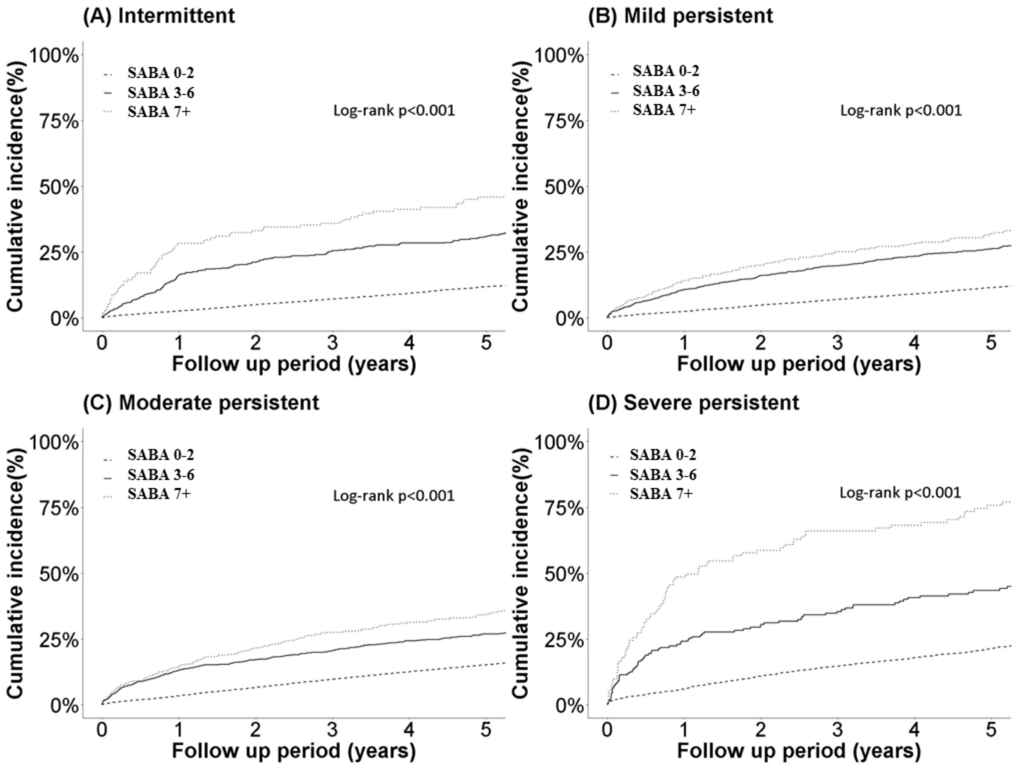

Supplementary Figure 1. Kaplan-Meier plot of the incidence of asthma exacerbation by baseline SABA use of non-ICS users with different severity: (A)intermittent, (B)mild persistent, (C)moderate persistent, and (D)severe persistent.

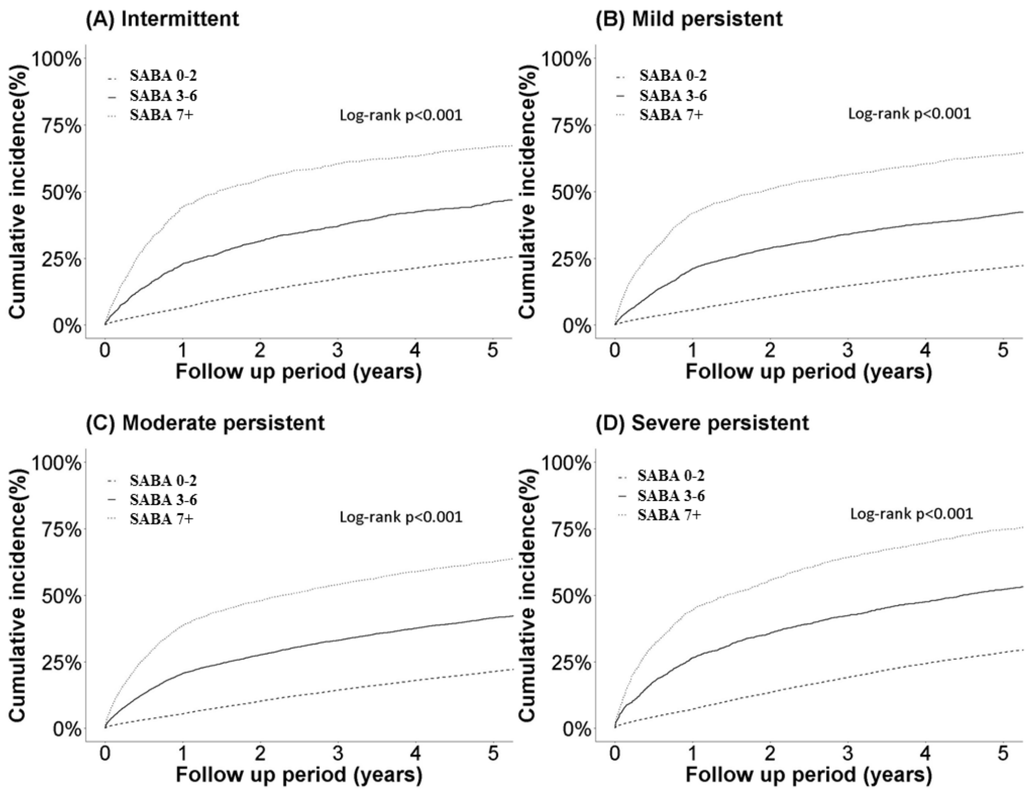

Supplementary Figure 2. Kaplan-Meier plot of the incidence of asthma exacerbation by baseline SABA use of ICS users with different severity: (A)intermittent, (B)mild persistent, (C)moderate persistent, and (D)severe persistent.

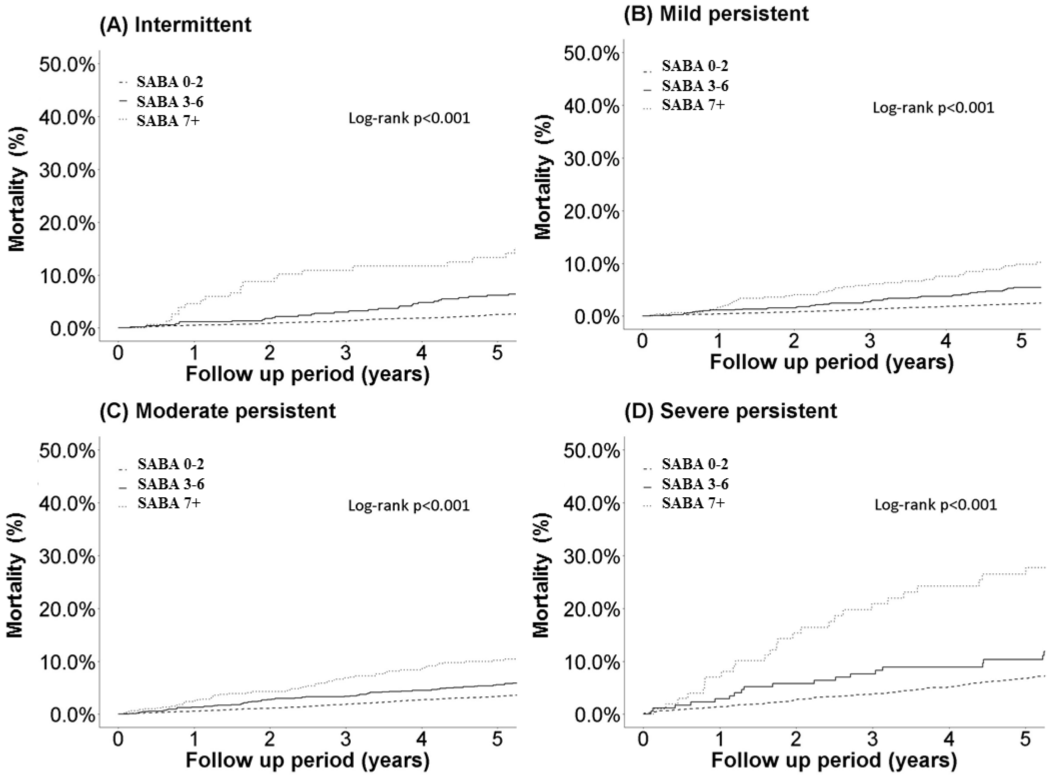

Supplementary Figure 3. Kaplan-Meier plot of mortality by baseline SABA use of non-ICS users with different severity: (A)intermittent, (B)mild persistent, (C)moderate persistent, and (D)severe persistent.

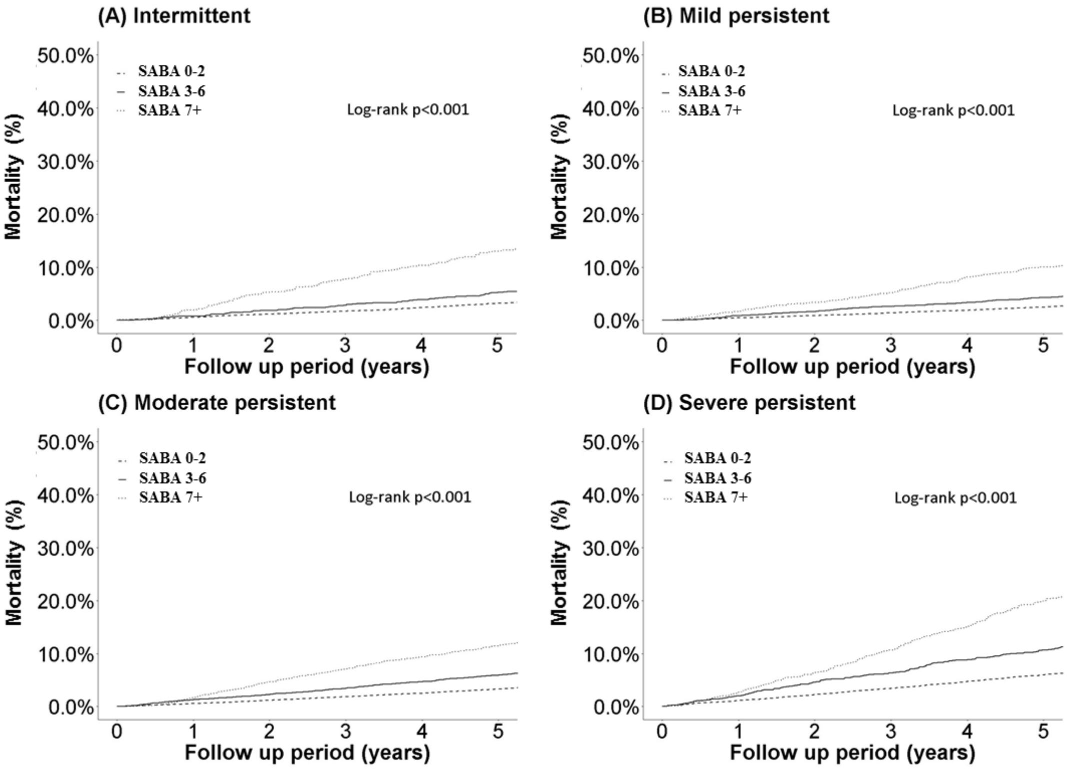

Supplementary Figure 4. Kaplan-Meier plot of mortality by baseline SABA use of ICS users with different severity: (A)intermittent, (B)mild persistent, (C)moderate persistent, and (D)severe persistent.
