# Supplementary material for: The prevalence and outcome of short-acting β2-agonists overuse in asthma patients in Taiwan
Source: NPJ Prim Care Respir Med. 2021 Apr 20;31:19. doi: 10.1038/s41533-021-00231-1 (PMC8058069; doi:10.1038/s41533-021-00231-1)
Supplement: Supplementary file 2 — Reporting Summary [file 41533_2021_231_MOESM2_ESM.pdf]

nature research

Corresponding author(s): Hao-Chien Wang

Last updated by author(s): Jan 6, 2021

Reporting Summary

Nature Research wishes to improve the reproducibility of the work that we publish. This form provides structure for consistency and transparency in reporting. For further information on Nature Research policies, see our Editorial Policies and the Editorial Policy Checklist.

Statistics

For all statistical analyses, confirm that the following items are present in the figure legend, table legend, main text, or Methods section.

n/a	Confirmed	
☐	☒	The exact sample size (*n*) for each experimental group/condition, given as a discrete number and unit of measurement
☐	☒	A statement on whether measurements were taken from distinct samples or whether the same sample was measured repeatedly
☐	☒	The statistical test(s) used AND whether they are one- or two-sided<br>*Only common tests should be described solely by name; describe more complex techniques in the Methods section.*
☐	☒	A description of all covariates tested
☐	☒	A description of any assumptions or corrections, such as tests of normality and adjustment for multiple comparisons
☐	☒	A full description of the statistical parameters including central tendency (e.g. means) or other basic estimates (e.g. regression coefficient) AND variation (e.g. standard deviation) or associated estimates of uncertainty (e.g. confidence intervals)
☐	☒	For null hypothesis testing, the test statistic (e.g. F, t, r) with confidence intervals, effect sizes, degrees of freedom and P value noted<br>*Give P values as exact values whenever suitable.*
☐	☒	For Bayesian analysis, information on the choice of priors and Markov chain Monte Carlo settings
☐	☒	For hierarchical and complex designs, identification of the appropriate level for tests and full reporting of outcomes
☐	☒	Estimates of effect sizes (e.g. Cohen's *d*, Pearson's *r*), indicating how they were calculated

Our web collection on statistics for biologists contains articles on many of the points above.

Software and code

Policy information about availability of computer code

Data collection	SAS version 9.4
Data analysis	SAS version 9.4

For manuscripts utilizing custom algorithms or software that are central to the research but not yet described in published literature, software must be made available to editors and reviewers. We strongly encourage code deposition in a community repository (e.g. GitHub). See the Nature Research guidelines for submitting code & software for further information.

Data

Policy information about availability of data

All manuscripts must include a data availability statement. This statement should provide the following information, where applicable:

- Accession codes, unique identifiers, or web links for publicly available datasets
- A list of figures that have associated raw data
- A description of any restrictions on data availability

National Health Insurance Research Database (NHIRD) are derived from the Bureau of National Health Insurance, Taiwan (BNHI).

Field-specific reporting

Please select the one below that is the best fit for your research. If you are not sure, read the appropriate sections before making your selection.

☒ Life sciences ☐ Behavioural & social sciences ☐ Ecological, evolutionary & environmental sciences

For a reference copy of the document with all sections, see nature.com/documents/nr-reporting-summary-flat.pdf

Life sciences study design

All studies must disclose on these points even when the disclosure is negative.

Sample size	218,039 patients
Data exclusions	Patients were excluded for the following reasons: (1) age <12 or >100 years, (2) unknown demographic data, or (3) history of tuberculosis, bronchitis or other respiratory disease before entering the P4P asthma program.
Replication	The study population comprised patients aged 12 to 100 years old who were enrolled in the Taiwan P4P asthma program between 2001 and 2015.
Randomization	We used a propensity score-matched cohort study to minimize the effect of possible confounding factors. Propensity score match method was applied to identify two similar subgroup of the patients who collected 0 to 2 and ≥ 3 canister of SABA per year.
Blinding	We used a propensity score-matched cohort study to minimize the effect of possible confounding factors. Propensity score match method was applied to identify two similar subgroup of the patients who collected 0 to 2 and ≥ 3 canister of SABA per year.

Reporting for specific materials, systems and methods

We require information from authors about some types of materials, experimental systems and methods used in many studies. Here, indicate whether each material, system or method listed is relevant to your study. If you are not sure if a list item applies to your research, read the appropriate section before selecting a response.

Materials & experimental systems

n/a	Involved in the study	
☒	☐	Antibodies
☒	☐	Eukaryotic cell lines
☒	☐	Palaeontology and archaeology
☒	☐	Animals and other organisms
☐	☒	Human research participants
☐	☒	Clinical data
☒	☐	Dual use research of concern

Methods

n/a	Involved in the study	
☒	☐	ChIP-seq
☒	☐	Flow cytometry
☒	☐	MRI-based neuroimaging

Human research participants

Policy information about studies involving human research participants

Population characteristics	Asthma patients
Recruitment	Asthma patients aged 12 to 100 years old who were enrolled in the Taiwan P4P asthma program between 2001 and 2015.
Ethics oversight	The Ethical Committee of National Taiwan University Hospital approved the research (#201812069RIPC)

Note that full information on the approval of the study protocol must also be provided in the manuscript.

Clinical data

Policy information about clinical studies

All manuscripts should comply with the ICMJE guidelines for publication of clinical research and a completed CONSORT checklist must be included with all submissions.

Clinical trial registration	This is retrospective study.
Study protocol	This is retrospective study.
Data collection	This is retrospective study.

Outcomes

Severe exacerbation and all-cause mortality were measured during the follow-up period which started after the final day of the 12-month baseline period.
